# Factors Related to Self-Rated Health Among Community-Dwelling Older Adults

**DOI:** 10.3390/healthcare13030314

**Published:** 2025-02-04

**Authors:** Seoyoung Park, Se-Won Kang

**Affiliations:** 1 Department of Nursing, Gyeongsang National University, 33 Dongjin-ro, Jinju-si 52725, Republic of Korea; masternur@naver.com; 2 Department of Nursing, Dongseo University, 47 Jurye-ro, Sasang-gu, Busan 47011, Republic of Korea

**Keywords:** self-related health, community, older adults

## Abstract

Background/Objectives: This study explored factors influencing self-rated health (SRH) among community-dwelling older adults to better support its use in health screening and provide an alternative for older adults who may have difficulty working with lengthy assessment scales. Methods: The participants were 8379 individuals aged 65 years or older from the 2020 Elderly Survey. Data were collected in South Korea between September and November 2020. Descriptive statistics were calculated, and independent samples t-tests, a Kruskal–Wallis test, and weighted multiple regression analysis were conducted using IBM SPSS for Windows ver. 23.0. SRH factors, such as illness, daily living performance, nutritional status, depression, and cognitive impairment, were analyzed. Results: The greater the number of chronic conditions (β = −0.21, *p* < 0.001), the higher the dependence on activities of daily living and instrumental activities of daily living (β = −0.05, *p* = 0.002; β = −0.13, *p* < 0.001), the higher the depression score (β = −0.22, *p* < 0.001), the more severe the cognitive impairment (β = −0.04, *p* < 0.001), and the worse the SRH. Conclusions: Participants with high-risk medical conditions, such as cancer, stroke, and depression, thought their health was poor. However, they did not consider hypertension, malnutrition, and abnormal BMI as significantly affecting their health status. Therefore, these factors should be considered when measuring SRH in older adults.

## 1. Introduction

Population aging is an important social phenomenon, and South Korea is expected to become a super-aged society by 2025, with those aged 65 or older accounting for 16.5% of the total population [1]. The prevalence of chronic medical conditions among older adults in Korea is high, with 84% affected by at least one, highlighting that although life expectancy has increased, the proportion of older adults suffering from chronic medical conditions is high [2]. Additionally, older adults tend to suffer from multiple conditions, making old age the stage of life with the highest health-related medical expenses [3]. In addition, the issue of older adults’ health is increasingly being seen as a social and national problem rather than just an individual or family one [4].

Health status is generally described in three areas: medical, functional, and self-assessed [5]. Self-rated health (SRH, also termed “subjective health status”) is an indicator that independently predicts mortality along with several specific health indicators known to predict mortality [6]. Moreover, international organizations, such as the World Health Organization (WHO), the Organization for Economic Co-operation and Development, and the European Union, recommend using SRH when comparing health status between countries [7].

Self-rated health refers to an individual’s evaluation or perception of their overall health, including their physical and mental health [8]. The SRH of older adults concisely expresses various aspects of their health status. It is therefore a core factor in old age, representing quality of life, and exhibits a close relationship with life satisfaction. Older adults’ SRH is a comprehensive evaluation of physical, physiological, psychological, and social aspects and is influenced by past and present objective health status, illness, medical care utilization status, and various aspects of life [9,10,11]. A review of previous studies on SRH reported that socioeconomic factors—such as educational level, household income, real estate ownership, and subjective economic circumstances—significantly affect subjective health status [12,13]. Additionally, various factors, such as social support, emotional states (e.g., depression), the presence or absence of geriatric illnesses, stress, and psychological and physical activities, have been found to affect SRH [14] and it has been shown to be influenced by objective health status, emotions, values, and attitudes [15]. The more positively a person perceives their health status, the more likely they are to not only improve their condition but also maintain physical and mental health and enhance social accessibility. Positive health status perception has been shown to positively impact the ability to perform daily life activities and exercise; thus, the better the SRH, the more positively it affects objective health status [16]. Research also indicates a relationship between SRH and chronic conditions, including cardiovascular disease [17]. In one study on the relationship between subjectively assessed health and the incidence of cardiovascular disease, once sociodemographic, lifestyle-related, and health-related variables were controlled for, the incidence of cardiovascular disease was found to be 3.3 times higher in participants who assessed their health as “bad” than in participants who responded “very healthy” [18]. In terms of the relationship with “death”, it was reported that the mortality risk within four years was 43% higher for older adults who responded “unhealthy” than for those who responded “healthy”. Previous studies have also demonstrated that older adults’ SRH is an important indicator of quality of life and successful aging and is influenced by a variety of different factors [19]. Subjective health perception among middle-aged and older adults may not always align with objective health status. However, SRH might serve as an independent indicator in evaluating actual health status for older adults’ health screening or follow-up in community or busy clinical settings.

We analyzed large-scale national data concerning older adults in the community, focusing on aspects of physical and mental health status previous studies showed to be significant, to comprehensively analyze the factors affecting SRH. Specifically, physical health status was analyzed by focusing on the relationships between major medical conditions related to mortality in old age, activities of daily living (ADLs), instrumental activities of daily living (IADLs), and nutritional status. Mental health status was analyzed based on the influence of depression and cognitive impairment. Socioeconomic and general characteristics—such as educational level, residential area, residential type, and economic status, which previous studies have shown affect older adults’ subjective health status—were used as moderator variables. Unlike previous studies, however, we focused on analyzing the number of chronic medical conditions in older adults and the influences of each of the major diseases selected in this study.

### Study Purpose

This study aimed to identify determinants of self-rated health (SRH) among community-dwelling older adults to better support its use in health screening and provide an alternative for older adults who may have difficulty working with lengthy assessment scales. Its specific objectives were as follows:

- to investigate differences in SRH according to participants’ general characteristics
- to examine differences in SRH according to physical and mental health status
- to identify the factors associated with SRH vis à vis physical and mental health status.

## 2. Materials and Methods

### 2.1. Study Design

This cross-sectional study analyzed previously collected data to identify factors affecting SRH among community-dwelling older adults.

### 2.2. Study Participants

The study focused on individuals aged 65 years or older, selecting 8379 participants from the 2020 Elderly Nationwide Survey in South Korea based on the following criteria: (1) those who responded to the survey independently without help from a spouse, child, or others; (2) those who responded to the survey without missing responses for this study’s dependent and independent variables; and (3) excluding those who scored less than 19 points (defined as severe cognitive impairment) on the Korean version of the Mini-Mental State Exam (MMSE-K) [20] or were diagnosed with dementia by a physician.

### 2.3. Data Collection

This study utilized part of the data collected through Korea’s 2020 Elderly Survey, a statutory survey administered every three years to investigate the living conditions and welfare needs of older adults in South Korea and provide basic data for establishing welfare policies for older adults.

The raw data survey targeted older adults aged 65 or older living in their own homes nationwide. The target sample was extracted by sampling the survey district and number of households in line with the stratified cluster sampling method.

The data were initially grouped by 17 cities and provinces across the country and then further divided into nine provincial regions by dong (neighborhood/district) and township. To ensure reliable results, the final sample was calculated using a proportional distribution method, which took into account the number of adults aged 65 years or older by stratum.

Data were collected through one-on-one interviews with trained investigators visiting participants’ homes between September and November 2020. The questionnaire was designed to minimize errors by using the tablet PC-assisted personal interview method, on-site data verification by investigators, input error verification through programming, and data accuracy verification through statistical processing.

The Elderly Survey data [21] used for the study were provided and utilized according to the data use procedures laid down by the Health and Welfare Data Portal.

### 2.4. Study Variables

#### 2.4.1. Self-Rated Health (SRH)

SRH refers to an individual’s current health status. It was measured on a five-point Likert scale ranging from 1 (very poor) to 5 (very good/excellent) using a single question: “How would you rate your overall health status in recent days?”

#### 2.4.2. Physical Health Status

Physical health status includes the following three categories: illness, daily living performance, and nutritional status.

1. Illness

The category “illness” includes the number of chronic conditions that have persisted for more than three months following diagnosis by a physician, the number of different drugs being taken owing to the condition, and the presence or absence of each of the following: ① hypertension, ② stroke, ③ ischemic heart disease, ④ diabetes mellitus, ⑤ chronic obstructive pulmonary disease (COPD)/asthma, ⑥ cancer, ⑦ chronic kidney disease, ⑧ chronic orthopedic conditions (arthritis, osteoporosis, back pain/sciatic neuralgia), ⑨ cataracts/glaucoma, and ⑩ gastroduodenitis/gastric ulcer. These ten are chronic conditions and constitute the leading causes of death or most frequently treated medical conditions among older adults in South Korea. According to WHO global health estimates, the first seven listed are older-adult-related medical conditions that are among the top ten leading causes of death in upper-middle- and high-income countries [22]. Three of the top ten conditions listed by the WHO (COVID-19, tuberculosis, and diarrheal illnesses) were excluded because of their temporary or very low incidence and as having little to do with causes of death in South Korea. The last three conditions on the list were added as they were among the most frequently treated chronic conditions for older adults in South Korea between 2019 and 2021 [23].

2. Daily living Performance

Participants’ daily living performance was measured using ADLs and IADLs. While ADLs are used as an indicator of a person’s functional status, IADLs are well-known as complex activities related to the ability to live independently in the community [24,25].

The ADLs scale comprises seven items that assess the independent abilities of moving around independently, feeding, dressing, personal hygiene, continence, and using the toilet independently [24]. The IADLs scale comprises 10 items that assess complex thinking skills, including transportation and shopping, managing finances, shopping and meal preparation, housecleaning and home maintenance, managing communication with others, and managing medications [25]. The participants were asked the following question: How much was other people’s help needed to carry out each of these over the past week? The response to each item was rated as “did not need it” (0 points), “partly needed it” (1 point), or “completely needed it” (2 points). The higher the score, the higher the dependence. When all items of the scale were rated as not needed, the participant was considered independent, whereas if even one item was rated as partly or completely needed, the participant was considered dependent.

3. Nutritional status

Nutritional status was measured using the Korean version of the Nutritional Screening Initiative DETERMINE checklist (NSI) and body mass index (BMI).

The NSI [26] is used to screen older adults’ nutritional health status and previous studies have confirmed the reliability of the Korean version of the scale [27]. It comprises 10 items with binary alternatives of “yes” and “no”. Each item has a set weight (1–4 points). The total score is calculated by summing the weights of the items answered with “yes”. The maximum score is 21 points; the higher the score, the more severe the nutritional risk. Depending on the total score, participants are classified as “good nutritional status” (0–2), “moderate nutritional risk” (3–5), and “high nutritional risk” (6 and higher). In this study, the scale’s reliability had a Cronbach’s alpha of 0.813.

BMI refers to a person’s weight in kilograms divided by the square of their height in meters (kg/m^2^), and BMI scores are classified as follows: less than 18.5, underweight; 18.5–22.9, normal; 23–24.9, pre-obese; and 25 or more, obese [28].

#### 2.4.3. Mental Health Status

Mental health status was measured based on depression and cognitive impairment levels.

1. Depression

Depression was measured using the Korean version of the short form of the Geriatric Depression Scale [29]. The original Geriatric Depression Scale is used worldwide to screen older adults for depression. The scale comprises 15 items with binary alternatives of “yes” and “no”. The score for each item is 1 point for “yes” and 0 points for “no” and the total score is calculated by summing the number of “yes” answers. If the total score based on a maximum of 15 points is 5 or more, the individual is considered at risk for depression [30,31]. In this study, this scale’s reliability had a Cronbach’s alpha of 0.890.

2. Cognitive impairment

Cognitive impairment was measured using the Korean version of the Mini-Mental State Exam (MMSE-K) [20] which studies have shown to be a valid instrument for screening cognitive impairment [32]. This scale comprises tasks, instructions, and scoring. The tasks include date and place orientation, registration of three objects, serial sevens, recall of three objects, naming, repeating a phrase, verbal commands, drawing, and two modified tasks. To allow for illiteracy, in our testing the tasks with written commands and sentence writing were modified to understanding the meaning of a sentence and a proverb (the reason for washing clothes and the meaning of “many a little makes a mickle”). Each task comprises 1–5 instructions, and each instruction is scored using binary alternatives (correct, 1 point; incorrect, 0 points). The maximum possible score is 30 points. The score ranges and classifications for community-dwelling older adults are as follows [20]: more than 24 points, normal; 20–23 points, mild cognitive impairment; and 19 points or less, severe cognitive impairment. If the individual was never educated or was illiterate, we added four points to the total score. Consequently, the score ranges can be applied regardless of age, sex, or educational level.

#### 2.4.4. Adjusted Variables

The adjusted variables were age, educational level, religion, residential area, marital status, living together, homeownership, annual income, smoking, drinking, visiting a physician, and hospitalization.

Old age was classified into early (65–74), middle (75–84), and late (85 years and older). Smoking was measured by asking “Do you currently smoke?”. Drinking was measured using a 6-point Likert scale (0, none; 1, less than once a month; 2, once a month; 3, twice a month; 4, once a week; and 5, more than twice a week) and by asking “How often have you had a drink in the past year?”. Visiting a physician was measured using one question with binary alternatives of “yes” or “no” as follows: “Have you visited a physician in the past month?”. Being hospitalized was measured using one question with binary alternatives of “yes” or “no” as follows: “Have you been hospitalized in the past year?”.

### 2.5. Ethical Considerations

The 2020 Elderly Survey was reviewed and approved by the Institutional Review Board (IRB) of the Korea Institute for Health and Social Affairs (Korea Institute for Health and Social Affairs IRB Review Results Notice No. 2020-36). Data collection for this survey was conducted by trained investigators after obtaining written consent from all participants. The data used in this study can be used by anyone through a simple approval process on the Microdata Integration Service website [21], and the participants cannot be identified because their personal information is not included.

### 2.6. Data Analysis

All data were analyzed using SPSS version 23.0 for Windows (IBM, Armonk, NY, USA).

(1) In accordance with practice guidelines, data cleaning was performed to ensure that the quality of the data were reliable [33].
(2) The participants’ general characteristics and physical and mental health status were presented as frequencies, percentages, means, and standard deviations. Differences in SRH according to these variables were analyzed using independent sample t-tests and the Kruskal–Wallis test as non-parametric methods. Post hoc analysis of the Kruskal–Wallis test was performed using Dunnett’s test.
(3) Weighted multiple regression was used to identify the factors associated with SRH.
(4) This study’s statistical significance level was *p* < 0.05.

## 3. Results

### 3.1. Differences in SRH According to Participants’ General Characteristics

Table 1 presents the participants’ general characteristics and the differences in SRH according to these characteristics. Notably, the participants’ average age was 72.6 years. Of the participants, 5476 (65.4%) were aged 65–74, 7390 (88.2%) did not smoke, 5150 (60.9%) did not drink, 5672 (67.7%) had visited the hospital outpatient clinic within the past month, and 518 (6.2%) had been hospitalized within the past year.

The SRH difference verification according to the participants’ characteristics revealed differences by age (χ^2^ = 613.63, *p* < 0.001) and educational level (χ^2^ = 599.52, *p* < 0.001). Additionally, the SRH of participants living in a household with a spouse or someone else was significantly higher than for those living alone (χ^2^ = 156.44, *p* < 0.001). The participants’ average annual income was 28,103 (±3877.15) won, and the SRH of participants with an above-average income was significantly higher than that of participants with incomes below the average (t = −15.33, *p* < 0.001). Participants who smoked (t = −7.52, *p* < 0.001) and did not drink alcohol at all had the lowest SRH (χ^2^ = 260.68, *p* < 0.001). The SRH of participants who had visited an outpatient clinic within the past month (t = 23.04, *p* < 0.001) or had been hospitalized within the past year (t = 11.14, *p* < 0.001) was significantly lower than that of those who had not.

### 3.2. Differences in SRH According to Physical and Mental Health Status

Table 2 presents the differences in SRH according to physical and mental health status.

For physical health status, ① of the 8379 participants, 1525 (18.2%) did not have a chronic condition, and the group as a whole averaged 1.8 (±1.43) such conditions; the greater the number, the more significantly lower their SRH (χ^2^ = 1759.24, *p* < 0.001). The average number of medications being taken was 1.7 (±1.42), and again, the more different medications being taken, the more significantly lower their SRH (χ^2^ = 1690.66, *p* < 0.001). Among the 10 conditions, cancer exhibited the lowest SRH (2.4 ± 0.89), and chronic orthopedic conditions showed the greatest SRH difference (t = 27.02, *p* < 0.001), while the smallest difference (t = 6.05, *p* < 0.001) was associated with gastroduodenitis/ulcers.

② Regarding daily living performance, the SRH of participants with dependent ADL (t = 25.96, *p* < 0.001) and IADL (t = 23.03, *p* < 0.001) was significantly lower.

③ Regarding nutritional health status, 21.5% of participants were in the NSI nutritional risk group and SRH significantly decreased as the risk increased (χ^2^ = 466.79, *p* < 0.001). Participants who were overweight (as assessed by their BMI) had the highest SRH (3.5 ± 0.77) and those who were underweight the lowest (3.0 ± 1.04).

For mental health status, the SRH difference was significantly lower in participants at risk of depression (t = 27.52, *p* < 0.001) and in those with mild cognitive impairment (t = 15.67, *p* < 0.001).

### 3.3. Factors Associated with SRH

Table 3 presents the results of the weighted multiple regression analyzing the factors related to SRH.

Regarding illness-related variables of physical health status, the numbers with chronic conditions (β = −0.21, *p* < 0.001), hypertension (β = 0.04, *p* = 0.001), stroke (β = −0.06, *p* < 0.001), ischemic heart disease (β = −0.04, *p* < 0.001), diabetes mellitus (β = −0.03, *p* = 0.003), COPD or asthma (β = −0.04, *p* < 0.001), cancer (β = −0.08, *p* < 0.001), chronic kidney disease (β = −0.05, *p* < 0.001), chronic orthopedic conditions (β = −0.05, *p* < 0.001), and gastroduodenitis or ulcer (β = 0.03, *p* = 0.002) were significant factors. Regarding the daily living performance variables, ADLs (β = −0.05, *p* = 0.002) and IADLs (β = −0.13, *p* < 0.001) were significant factors.

Regarding mental health status, depression (β = −0.22, *p* < 0.001) and cognitive impairment (β = −0.04, *p* < 0.001) were significant variables.

On the contrary, the number of medications taken (β = −0.04, *p* = 0.079), cataract or glaucoma (β = 0.01, *p* = 0.260), and abnormal BMI (β = −0.01, *p* = 0.148; β = 0.01, *p* = 0.353; β = 0.01, *p* = 0.176) did exhibit significant associations. Moreover, those classified by the NSI as being in the “higher risk” group reported higher SRH than those classified as “good” (β = 0.02, *p* = 0.048; β = 0.04, *p* < 0.001). The model’s adjusted R² value was 0.416.

## 4. Discussion

This study aimed to identify factors influencing SRH among community-dwelling older adults, with a specific focus on physical and mental health factors, such as illness, daily living performance, nutritional status, depression, and cognitive impairment. The results of this study reveal that higher numbers of chronic conditions (β = −0.21, *p* < 0.001) resulted in higher dependence on ADLs and IADLs (β = −0.05, *p* = 0.002; β = −0.13, *p* < 0.001), higher depression scores (β = −0.22, *p* < 0.001), more severe cognitive impairment (β = −0.04, *p* < 0.001), and worse SRH. Furthermore, SRH is evaluated differently, depending on the illness. Participants with conditions known to be high-risk, such as cancer and stroke, consider their health poor. Participants with conditions that are not recognized or are considered treatable and of low risk to life, such as gastroduodenitis/ulcer, cataract/glaucoma and hypertension, consider their health good and exhibit high SRH. Abnormal BMI does not significantly impact SRH, and the higher the risk of malnutrition, the better the SRH. In fact, this may mean that older adults cannot associate their health with nutritional status or there may be differences between the tools used and the controlled variables. Therefore, additional analyses will be required in the future.

In physical health status, this results of the study indicate that the greater the number of chronic conditions, the higher the risk of those known conditions, the greater the impact on subjective health. In Kwak et al.’s study [17], analysis of the relationship between illness and subjective health reveals that diabetes, heart disease, gastric ulcer, and chronic obstructive pulmonary disease are subjectively associated with poor subjective health. In Hu and Lu’s study [34], chronic conditions such as hypertension, heart disease, diabetes, cerebrovascular disease, and gastroenteritis are related. Although this results of this study differ from those of others in relation to hypertension, gastric ulcers, and gastroenteritis, they do suggest that participants’ chronic conditions affect their subjective health. Additionally, as in Confortin et al.’s study [35], the number of chronic conditions an individual suffers from affects their subjective health, and is therefore a significant variable. It can be argued that illnesses among older adults cause physical and emotional changes, further affecting their daily activities and quality of life, and thereby directly affecting an individual’s subjective evaluation of their overall health. The higher the dependence on ADLs and IADLs, the worse the individual’s self-evaluation of their health. These results are consistent with Hwang and Kim’s finding [36] regarding older adults’ health-related quality of life: if no difficulties exist in exercise ability, self-management, and daily life, SRH is perceived positively. This aspect should be considered when evaluating SRH. Additionally, to improve subjective health evaluation, older adults’ ability to perform daily life activities should be improved by maintaining and improving their physical strength. This will in turn lead to improvements in the emotional and social aspects of their lives.

In terms of mental health status, depression is the main factor affecting SRH, which is consistent with a previous study by Lee [37]. As SRH includes both objective and subjective health status as perceived by the individual, a possible interpretation is that, as the depression level increases, individuals tend to evaluate their SRH negatively because they are influenced by personal emotions, values, and attitudes. Cognitive function is another major factor affecting SRH, supporting the results reported by Hwang and Kim [36]. Seemingly, the more that older adults perceive their own health status as poor, the lower their cognitive function becomes. Older adults are characterized by declining physical conditions over time, such as skeletal muscle mass and weight, which are likely to negatively impact cognitive function. Enhancing older adults’ perception of their health status and improving their mental and physical health status can help evaluate their SRH.

The results of this study may be the result of the characteristics of the elderly in Korea. Current Korean welfare policies, in addition to considering the promotion of physical health, need to focus on measures to improve emotional and psychological stability, such as by combating depression in the elderly. The results of this study, if acted upon, should improve the SRH of the elderly in Korea. In addition, through additional research on the various factors affecting SRH, a more detailed and specific guide for appropriate health management of older adults can be developed.

### Study Limitation

This study has several limitations. First, it relies exclusively on previously collected data. Second, as this was a cross-sectional study, limitations exist in inferring causal relationships between the variables over time. Future longitudinal studies addressing these limitations, such as by adding variables related to SRH in community-dwelling older adults, are required. Finally, although nutritional status was significant in the basic statistical analysis, it was not identified as a major factor in the multiple regression analysis after controlling for variables. This suggests that the tools used to measure nutritional status may have limitations, and future studies should consider using improved or alternative measurement methods for further validation.

## 5. Conclusions

Our study shows meaningful factors associated with the SRH of older adults. Having more chronic diseases, dependent IADL, and depression were strong influencing factors of low SRH. When their physical and mental functions render daily life activities difficult, they perceive their health as poor and evaluate their SRH as low. However, they may be unable to relate these perceptions to their health if they have conditions that do not cause specific symptoms, such as hypertension, or if they are malnourished or have a BMI outside the normal range. Therefore, these factors should be considered when using SRH to measure the health status of older adults. In addition, the possibility of depression or cognitive problems should be considered if older adults without physical problems have lower SRH. The results of this study clearly show that when developing health education and health policies for community-dwelling older adults, the major factors affecting SRH—such as chronic conditions, ADLs and IADLs, depression, and cognitive impairment—need to be taken into consideration. We hope that our findings, combined with further research on the various factors affecting SRH, will lead to the provision of detailed and specific health management guidelines.

## Figures and Tables

**Table 1:** Differences in self-related health according to participants’ characteristics.

Variable		*n* (%)	Self-Rated Health	
			Mean ± SD	t(p) or χ^2^
Sex	Male	3321 (39.6)	3.4 ± 0.84	0.07 −0.942
	Female	5058 (60.4)	3.4 ± 0.84	
Age (years)	65–74 ^a^	5476 (65.4)	3.6 ± 0.78	613.63 ** (<0.001) a > b > c
	75–84 ^b^	2544 (30.4)	3.1 ± 0.86	
	≥85 ^c^	359 (4.3)	3.0 ± 0.88	
	Mean ± SD	72.6 ± 6.10		
Educational level	≤Primary education ^a^	3344 (39.9)	3.2 ± 0.85	599.52 ** (<0.001) a > b > c
	Secondary education ^b^	4550 (54.3)	3.6 ± 0.76	
	Higher education ^c^	485 (5.8)	3.8 ± 0.89	
Religion	No	3395 (40.5)	3.5 ± 0.77	7.88 (<0.001)
	Yes	4984 (59.5)	3.4 ± 0.88	
Residential area	Seoul/satellite cities ^a^	2056 (24.5)	3.5 ± 0.87	16.81 ** (<0.001) a > b,c
	Metropolitan cities ^b^	2621 (31.3)	3.4 ± 0.81	
	Provincial cities ^c^	3702 (44.2)	3.4 ± 0.84	
Spouse	No	3192 (38.1)	3.2 ± 0.86	−14.17 (<0.001)
	Yes	5187 (61.9)	3.5 ± 0.81	
Living together	Living alone ^a^	2450 (29.2)	3.2 ± 0.87	156.44 ** (<0.001) b > c > a
	Living with a spouse only ^b^	4487 (53.6)	3.5 ± 0.81	
	Living with someone ^c^	1442 (17.2)	3.4 ± 0.85	
Homeownership	Homeowner	6760 (80.7)	3.5 ± 0.81	8.76 (<0.001)
	Other	1619 (19.3)	3.2 ± 0.93	
Annual income *	<2810	5471 (65.3)	3.3 ± 0.85	−15.33 (<0.001)
	≥2810	2908 (34.7)	3.6 ± 0.79	
	Mean ± SD	2810.3 ± 3877.15		
Smoking	No	7390 (88.2)	3.4 ± 0.85	−7.52 (<0.001)
	Yes	989 (11.8)	3.6 ± 0.76	
Drinking	No ^a^	5105 (60.9)	3.3 ± 0.88	260.68 ** (<0.001) e > b,c,d,f > a
	<Once a month ^b^	450 (5.4)	3.5 ± 0.79	
	Once a month ^c^	748 (8.9)	3.6 ± 0.75	
	Once or twice a month ^d^	787 (9.4)	3.6 ± 0.70	
	Once a week ^e^	688 (8.2)	3.7 ± 0.70	
	>Once a week ^f^	600 (7.2)	3.6 ± 0.72	
Visited a physician in the past month	No	2707 (32.3)	3.7 ± 0.72	23.04 (<0.001)
	Yes	5672 (67.7)	3.3 ± 0.86	
Hospitalized in the past year	No	7861 (93.8)	3.4 ± 0.82	11.14 (<0.001)
	Yes	518 (6.2)	2.9 ± 1.01	

* Korean won, ten thousand, 2810: participants’ average annual income. ** Kruskal–Wallis test; post hoc: Dunnett’s test. Note. SD: standard deviation. The indications of a, b, c, d, e, and f are the categories of variables.

**Table 2:** Differences in self-related health according to physical and mental health status.

Variable		*n* (%)	Self-Rated Health	
			Mean ± SD	t(p) or χ^2^
Physical health status				
① Illness				
Chronic conditions (number)	0 ^a^	1525 (18.2)	3.9 ± 0.58	1759.24 ** (<0.001) a > b > c > d > e > f
	1 ^b^	2567 (30.6)	3.6 ± 0.69	
	2 ^c^	2260 (27.0)	3.3 ± 0.78	
	3 ^d^	1144 (13.7)	3.0 ± 0.89	
	4 ^e^	512 (6.1)	2.8 ± 0.87	
	≥5 ^f^	371 (4.4)	2.4 ± 0.78	
	Mean ± SD	1.8 ± 1.43		
Taking medicine (number of types)	0 ^a^	1680 (20.1)	3.9 ± 0.61	1690.66 ** (<0.001) a > b > c > d > e > f
	1 ^b^	2612 (31.2)	3.6 ± 0.70	
	2 ^c^	2161 (25.8)	3.3 ± 0.79	
	3 ^d^	1182 (14.1)	3.0 ± 0.89	
	4 ^e^	457 (5.5)	2.6 ± 0.84	
	≥5 ^f^	287 (3.4)	2.4 ± 0.80	
	Mean ± SD	1.7 ± 1.42		
Hypertension	No	3666 (43.8)	3.6 ± 0.79	15.76 (<0.001)
	Yes	4713 (56.2)	3.3 ± 0.85	
Stroke	No	8095 (96.6)	3.4 ± 0.82	14.44 (<0.001)
	Yes	284 (3.4)	2.6 ± 0.93	
Ischemic heart disease	No	8009 (95.6)	3.4 ± 0.83	14.10 (<0.001)
	Yes	370 (4.4)	2.8 ± 0.88	
Diabetes mellitus	No	6455 (77.0)	3.5 ± 0.80	18.58 (<0.001)
	Yes	1924 (23.0)	3.1 ± 0.88	
COPD or asthma	No	8148 (97.2)	3.4 ± 0.83	10.79 (<0.001)
	Yes	231 (2.8)	2.9 ± 0.80	
Cancer	No	8242 (98.4)	3.4 ± 0.83	14.66 (<0.001)
	Yes	137 (1.6)	2.4 ± 0.89	
Chronic kidney disease	No	8303 (99.1)	3.4 ± 0.84	7.25 (<0.001)
	Yes	76 (0.9)	2.7 ± 0.92	
Chronic orthopedic conditions	No	6250 (74.6)	3.6 ± 0.78	27.02 (<0.001)
	Yes	2129 (25.4)	3.0 ± 0.87	
Cataract or glaucoma	No	7945 (94.8)	3.4 ± 0.83	6.98 (<0.001)
	Yes	434 (5.2)	3.1 ± 0.87	
Gastroduodenitis or ulcer	No	8018 (95.7)	3.4 ± 0.83	6.05 (<0.001)
	Yes	361 (4.3)	3.2 ± 0.89	
② Daily living performance				
ADLs	Independent	8154 (97.3)	3.5 ± 0.79	25.96 (<0.001)
	Dependent	225 (2.7)	1.9 ± 0.91	
IADLs	Independent	7781 (92.9)	3.5 ± 0.78	23.03 (<0.001)
	Dependent	598 (7.1)	2.5 ± 1.03	
③ Nutritional health status				
NSI	Good ^a^	6574 (78.5)	3.5 ± 0.78	466.79 ** (<0.001) a > b > c
	Moderate risk ^b^	1224 (14.6)	3.1 ± 0.91	
	High risk ^c^	581 (6.9)	2.9 ± 0.95	
BMI *	Normal ^a^	3110 (38.6)	3.4 ± 0.86	76.01 ** (<0.001) c > a,d > b
	Underweight ^b^	162 (2.0)	3.0 ± 1.04	
	Pre-obese ^c^	2739 (34.0)	3.5 ± 0.77	
	Obese ^d^	2042 (25.4)	3.4 ± 0.86	
Mental health status				
Depression	Not at risk	6684 (79.8)	3.6 ± 0.76	27.52 (<0.001)
	At risk	1695 (20.2)	2.9 ± 0.92	
Cognitive impairment	None	6459 (77.1)	3.5 ± 0.82	15.67 (<0.001)
	Suspected	1920 (22.9)	3.2 ± 0.84	

* Except those who cannot measure height and weight or do not know. ** Kruskal–Wallis test; post hoc: Dunnett’s test. Note. SD: standard deviation; COPD: chronic obstructive pulmonary disease; ADLs: activities of daily living; IADLs: instrumental activities of daily living; NSI: nutrition screening initiative; BMI: body mass index (kg/m^2^). The indications of a, b, c, d, e, and f are the categories of variables.

**Table 3:** Factors associated with self-rated health by weighted multiple regression.

Variable		β	SE	*p*
Physical health status				
1 Illness				
Number of chronic conditions		−0.21	0.014	<0.001
Number of taking medicine		−0.04	0.013	0.079
Condition (ref. = none)				
Hypertension		0.04	0.019	0.001
Stroke		−0.06	0.042	<0.001
Ischemic heart disease		−0.04	0.038	<0.001
Diabetes mellitus		−0.03	0.021	0.003
COPD or asthma		−0.04	0.043	<0.001
Cancer		−0.08	0.059	<0.001
Chronic kidney disease		−0.05	0.072	<0.001
Chronic orthopedic conditions		−0.05	0.022	<0.001
Cataract or glaucoma		0.01	0.034	0.260
Gastroduodenitis or ulcer		0.03	0.036	0.002
2 Daily living performance				
ADLs		−0.05	0.017	0.002
IADLs		−0.13	0.007	<0.001
3 Nutritional status				
NSI (ref. = good)	Moderate risk	0.02	0.023	0.048
	High risk	0.04	0.032	<0.001
BMI (ref. = normal)	Underweight	−0.01	0.050	0.148
	Pre-obese	0.01	0.017	0.353
	Obese	0.01	0.019	0.176
Mental health status				
Depression		−0.22	0.003	<0.001
Cognitive impairment (ref. = none)	Suspected	−0.04	0.019	<0.001
R^2^, 0.419 Adjusted R^2^, 0.416 Change R^2^, 0.210 F(p), 160.35 (<0.001)				

Note. COPD: chronic obstructive pulmonary disease; ADLs: activities of daily living; IADLs: instrumental activities of daily living; NSI: nutrition screening initiative; BMI: body mass index (kg/m^2^), SE: standard error. Adjusted variables: age, educational level, religion, residential area, marital status, living together, homeownership, annual income, smoking, drinking, visiting a physician, and being hospitalized.

## Data Availability

The data presented in this study are available in [Korean Statistics Promotion Institute] at [https://mdis.kostat.go.kr/index.do] (accessed on 1 August 2024).
